# Dry immersion as a model of deafferentation: A neurophysiology study using somatosensory evoked potentials

**DOI:** 10.1371/journal.pone.0201704

**Published:** 2018-08-22

**Authors:** Blandine Acket, Liubov Amirova, Angelique Gerdelat, Pascal Cintas, Marc-Antoine Custaud, Anne Pavy-LeTraon

**Affiliations:** 1 Neurology Department, Centre Hospitalier Universitaire de Toulouse, Hôpital Purpan, Toulouse, France; 2 Institute of Biomedical Problems, Russian Academy of Sciences, Moscow, Russia; 3 University of Angers, Medicine Faculty, Mitovasc Laboratory, UMR CNRS 6015, INSERM, Angers, France; 4 Clinical Research Center, CHU d'Angers, France; 5 INSERM U1048, Equipe 8, Toulouse, France; Universite de Caen Normandie, FRANCE

## Abstract

**Introduction:**

Dry immersion is a ground-based experiment simulating the effects of weightlessness, and it is a model of acute symmetrical bilateral deafferentation. This exploratory study aimed to investigate the effects of three days of dry immersion (DI) on sensory thresholds and the functioning of lemniscal pathways, assessed by somatosensory evoked potentials (SEPs).

**Methods:**

Twelve healthy male volunteers (32+/-4.8 years) participated in the study. Sensory thresholds and SEPs of the tibial nerve of both limbs were recorded before (D-1) and on the third day of dry immersion (D3).

**Results:**

Sensory thresholds significantly decreased on D3 (-20.75 +/-21.7%; z = -2.54; p = 0.0109 on the right side and -22.18+/-17.28%; z = -3.059; p = 0.002 on the left side). The amplitude of P40 responses did not differ between D-1 and D3. Latencies of all central responses until P30 were shortened on D3 (N21 right:-0.57+/-0.31; z = -3.06; p = 0.002; N21 left -0.83+/-0.53; z = -2.94; p = 0.003; P30 right: -1.26+/-1.42; z = -3.059; p = 0.002; P30 left: -1.11+/-1.55; z = -2.27; p = 0.02)

**Conclusion:**

Three days of dry immersion can induce hyperexcitability of lemniscal pathways.

**Significance:**

This may be explained by a change in the expression of membrane channels and/or medullar plasticity and/or hypersensitization of peripheral sensory receptors induced by this acute deafferentation. Additional studies are needed to further elucidate the mechanisms.

## Introduction

### Weightlessness and dry immersion

Studies of the effects of space environment on the human body have been carried out for many years: when the body is exposed to prolonged periods of weightlessness, the effects include significant loss of bone and muscle mass, strength, cardiovascular and sensory-motor deconditioning and hormonal and metabolic changes. Taking into account the limited number of flight opportunities and the difficulties encountered in performing in-flight experiments, ground-based experiments simulating the effects of weightlessness are used to get a better understanding of the mechanisms of physiological adaptation. They are also used to design and validate countermeasures (preventive methods developed to reduce or eliminate the deconditioning associated with prolonged weightlessness).

**Fig 1:**
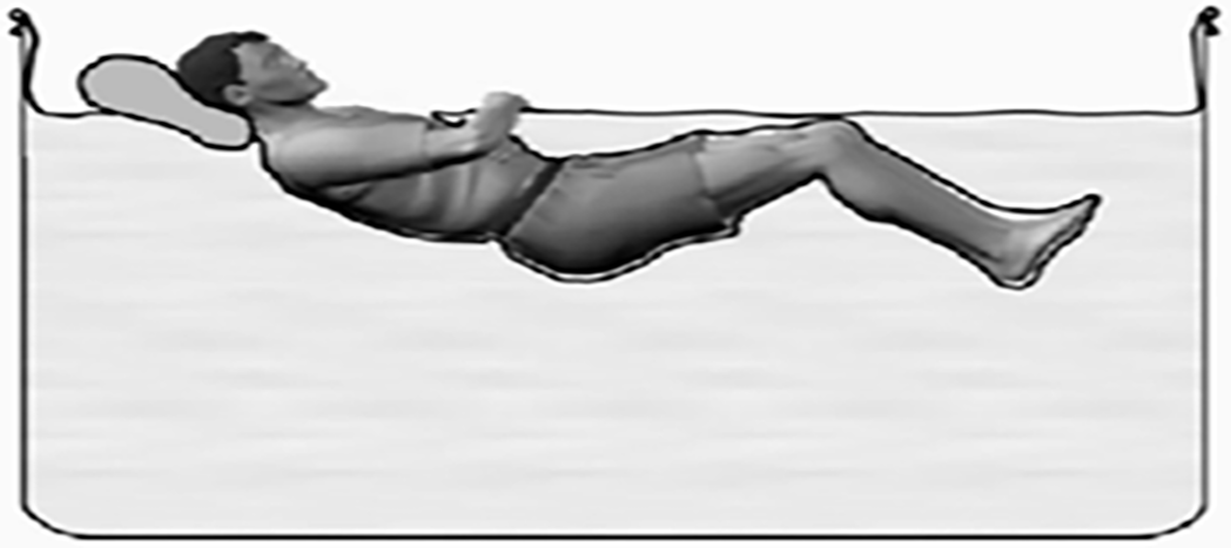
Dry immersion method: The subject is immersed up to the neck and separated from the water with a waterproof fabric.

Dry immersion is a ground-based model used to simulate microgravity effects. The subject is immersed up to the neck in thermoneutral water. However since he is separated from the water with an elastic waterproof fabric (Fig 1),the immersed subject is freely suspended in the water mass, but remains dry.

Different studies have been conducted using this technique lasting from 10 hours up to 56 days [1, 2]. The long-term effects of dry immersion are compared with those of bed rest and actual space flight.

For a relatively short period, as in bed rest, the model can faithfully reproduce most of the physiological effects of actual microgravity, including centralization of body fluids, support-unloading, and hypokinesia. Unlike bed rest, dry immersion provides a unique opportunity to study the physiological effects of the lack of a supporting structure for the body (‘supportlessness’)[3].

Consequently, dry immersion offers a unique opportunity to study the effects of the bilateral abolition of sensory afferents in humans.

The central nervous system integrates sensory afferents (vision, vestibular system, cutaneous and musculo-tendinous afferents) to adjust posture and coordinate movements. During dry immersion, the usual mechanical constraints are no longer applied to the plantar soles and back. The loss of axial tone is one of the earliest effects of dry immersion[3]. It is triggered by supportlessness and the decrease of sensory afferents. Changes in motor control that are induced by dry immersion include spinal hyperreflexia, suppression of tonic motor neuron activities and alteration of the pattern of motor neuron recruitment, sensory and motor ataxia, and alterations in strategies for movement control[3]. Pathophysiological mechanisms underpinning these changes are not well understood, and the precise involvement of the central nervous system in sensory processing, sensorimotor integration and motor control as a result of these environmental modifications remains unclear.

### Plasticity resulting from deafferentation

Environmental changes cause central nervous system plasticity that results in functional and structural modifications. The amputation of a limb is a model of chronic deafferentation. Several imaging studies have reported a reorganization of the somatosensory cortex. People with arm or hand amputation present a shift of the mouth into the hand in the primary somatosensory cortex (S1) [1, 2, 4]. Short-term deprivation of sensory input by ischemic nerve block or by locoregional anesthesia are models of acute functional deafferentation [5–7]. Werhahn and colleagues found a focal increase in excitability in the hand motor representation contralateral to the deafferented cortex [6]. They also found a rapid improvement in tactile acuity and changes in cortical processing for the left hand during cutaneous anesthesia of the right hand [7]. Both could be influenced by transcallosal interactions and GABAergic transmission [7].

A study using regional anesthesia emphasized the occurrence of illusions similar to those observed in phantom limb pain and suggests that anesthesia could trigger similar cerebral plasticity mechanisms [5].

In healthy subjects, temporary functional deafferentation improves sensorimotor functions of the contralateral homonymous regions or neighboring ipsilateral regions. Similar results have been obtained following stroke. Changes in the interhemispheric balance, primary motor cortex plasticity and ipsilateral S1 cortical reorganization, could be involved [8, 9].

### Hypothesis

Dry immersion is a unique model of acute symmetrical bilateral deafferentation. This study aimed at elucidating the adaptation capacity of the peripheral and central lemniscal system following precociously acute and temporary bilateral deafferentation induced by 3 days of dry immersion in healthy subjects. We formulated the hypothesis that this acute deafferentation could induce early somatosensory cortex reorganization. The underlying mechanisms could involve changes in intracortical inhibition or facilitation levels. This functional rapid cerebral plasticity could be assessed by modifications of somatosensory evoked potentials (SEPs).

## Methods

This was an open, single-center prospective study. Twelve healthy male subjects participated in the study. This study took place at the MEDES Space clinic located in the Toulouse hospital. It was supported by the French Space Agency (CNES) and coordinated by MEDES (Institute of Space Medicine and Physiology). The study was approved by the Ethics Committee (CPP Sud-Ouest Outre-Mer I) and the French Health Authorities.

### Subjects

#### Inclusion criteria

The subjects were healthy male volunteers, aged from 20 to 45, neither over- nor underweight, with a BMI (weight Kg/height m2) between 20 and 26, height between 158 and 190 cm, with no personal or family history of chronic or acute disease or psychological disturbance which could affect the physiological data and/or create a risk for the subject during the experiment. They had fitness level assessments (if age < 35 years: 35 ml/min/kg < VO2max < 60ml/min/kg; if age > 35 years: 30 ml/min/kg < VO2max < 60ml/min/kg).

They were active and free from orthopedic, musculoskeletal and cardiovascular disorders, non-smokers, with no alcohol or drug dependence and no ongoing medical treatment. They all signed informed consent, and were free from all engagements during the study.

#### Exclusion criteria

Exclusion criteria were a history of any disease or risk factor for thrombosis, a subject already participating or in the exclusion period of a clinical research study, refusal to give permission to contact his general practitioner, incarcerated persons, a subject who, in the judgment of the investigator, was likely to be non-compliant during the study or unable to cooperate because of a language problem or poor mental development, or a subject under guardianship or trusteeship.

### Methods

#### Dry immersion

The acute effects of dry immersion become apparent from the first 12 hours [3]. Changes in motor control that are induced by dry immersion are reproduced with three to seven days of dry immersion [10, 11].

This three-days duration was determined by the French Space Agency. This three days experiment included several protocols in different domains (cardiovascular, muscular…). The choice of three days was adapted to the different experimental tasks. The length was chosen in order to be long enough to trigger the effects of supportlessness but not too long to improve tolerance of the experiment by volonteers.

Subjects were hospitalized at the MEDES Space clinic. The sensory threshold was assessed before the first day of dry immersion and at the end of the third day.

The dry immersion experiment was performed in a specially designed bath filled with tap water. A special high-elastic waterproof fabric was attached to a metal rim around the external border of the bath. The fabric area considerably exceeded the area of the water surface. The bath had a built-in lift for lowering and raising the subject. The subject, dressed in a comfortable athletic suit, was placed on the waterproof fabric after the fabric had first been covered with a cotton sheet for hygiene reasons. The subject was lowered slowly into the water on the lift and his body was gradually covered with the folds of the fabric together with the water they contained. The fabric was thin and of sufficient area to allow the subject to appear to be “freely suspended" in the water, under conditions that are similar to a complete lack of structural support (Fig 1). The subjects were permitted to put their hands out to work with a computer, eat, read and perform experimental tasks. The water temperature was regulated automatically. It was set at 32°C to 34.5°C (thermoneutral), and was adjusted for comfort within these limits at the subject’s request. During the whole hospitalisation phases, diet was monitored.Subjects received three principal meals (breakfast, lunch and dinner) and one snack per day (afternoon). The meals was defined by the MEDES nutritionist, provided by Toulouse Hospital and delivered to the subjects by the health assistants under the supervision of the MEDES nutritionist. The main nutritional aspects are recalled hereafter: Coffee, tea, and alcohol were forbidden.

- Liquid intake:The liquid intake will be between 35 and 50 ml/Kg/day (total water taking into account beverages and food). Coffee, tea, and alcohol were forbidden.
- Caloric intake:On the first day of hospitalization, the basal metabolic rate (BMR) was calculated in kcal/day using the WHO equation:

Age<30y: BMR = 15.3 x body weight (kg) + 679

Age>30y: BMR = 11.6 x body weight (kg) + 879

Volunteers ate their meal on a tray without getting out from their bath.

Urination was done in a bedpan in the bath. For showering and defecation, once a day, the volunteer was lifted out of the bath to take a shower and to go to the toilet (lying down at-6°, low head).

The air temperature was approximately 24°C, in order to maintain the heat balance when the subject was raised from the bath. The subject remained under constant medical observation 24 hours a day. For physical examinations and visual inspections of the skin, the folds of fabric could be moved apart without substantially changing the experimental conditions.

#### Evaluation

Our tests were always done at the beginning of the afternoon. Measurements of SEPs were done just following dry immersion. Patients came out of their dry immersion ten minutes before at a maximum.Skin temperature was not checked at the time of our tests but the tympanic temperature of the patients was controlled twice a day and did not significantly vary. Tests were done in a room which was adjacent to the DI room. This room temperature was controlled and did not changed. Hydratation and liquid balance of the volunteers were controlled

#### Sensory threshold

For sensory threshold assessment and SEP measurements we used a Dantec Natus device equipped with a headrest, with 3 channels linked to a laptop using Keypoint software.

We used the stimulator to assess the sensory threshold, applying it to the tibial nerve at the ankle and progressively increasing the intensity of the current until the subject felt it. This test was done twice in order to verify to reproducibility of the value. Then we noted this intensity in mA.

#### Somatosensory evoked potentials

SEPs consist of a series of waves that reflect sequential activation of neural structures along the somatosensory pathways. SEPs are elicited by electrical stimulation of peripheral nerves and are recorded by electrodes placed over the scalp (cup electrodes), spine, and peripheral nerves proximal to the stimulation site. The effect of dry immersion has been studied by the changes in latency and size of cortical responses.

We used a bipolar stimulator, and the intensity of the stimulation was fixed at twice the sensory threshold, and it was sufficient to be above the motor threshold triggering movements of the first toe. We stimulated the tibial nerve at the ankle with a 3Hz frequency and the length of the stimulus was 0.2 ms.

We used a bipolar recording with two cup electrodes (active-reference) placed at these sites:

- popliteal recording: the active was placed over the route of the tibial nerve at the popliteal fossa, exactly 4 to 6 cm over the popliteal fold, at equal distance between tendons of the semimembranosus and semitendinosus muscles within, and the biceps femoris tendon outside, and the reference was glued over the head of the fibula.
- medullar recording: the active electrode was placed at the first lumbar level and the reference was placed just over the umbilicus. To ensure placement of electrodes at the same place, both before and after DI, a mark was drawn on the skin with a marker at L1.
- Scalp recording: The active was placed at C’z, two centimeters behind Cz (Cz was located per the 10–20 international system) and the reference was located on the contralateral lobule of the ear.

**Fig 2:**
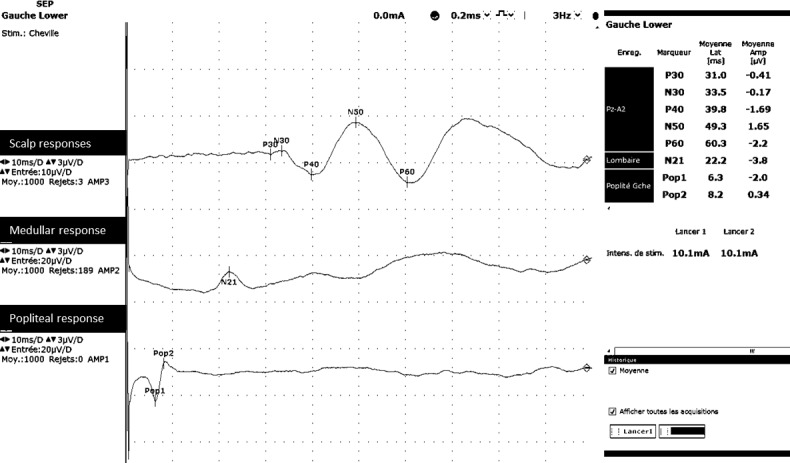
Drawings of raw typical exemple of evoked response potentials.

The impedance of each electrode was checked before beginning SEPs recording. For each set, 500 responses were averaged and 2 sets were performed on both sides. The bandwidth was between 3 and 3000 Hz. The analysis window was 100 ms (10 ms/division). Sampling rate was 48kHz. (Fig 2).

#### Study design

At baseline and after three days of DI immersion, we evaluated sensory thresholds on both sides and registered the SEPs following tibial nerve stimulation on both sides.

#### Statistical analysis

Based on previous studies, we focused on the Cz electrodes (references). The peak latencies at Cz were identified in the N21, N30, P40, N50, and P60 components, and peak amplitudes were measured for P40 (baseline to P40), N50 (P40-N50), and P60 (N50-P60). Data were separately submitted to a Wilcoxon rank test comparing the following data

- sensory threshold at baseline and after DI
- amplitude of P40 at baseline and after DI
- latencies of the following responses: popliteal, N21, P30, P40, N50, P60 at baseline and after DI
- conduction time pop-N21, N21-P30, N21-P40 at baseline and after DI

Correlations between sensory thresholds variation and the changes of the latencies before/after DI were investigated using the Pearson test.

## Results

### Sensory thresholds

**Fig 3:**
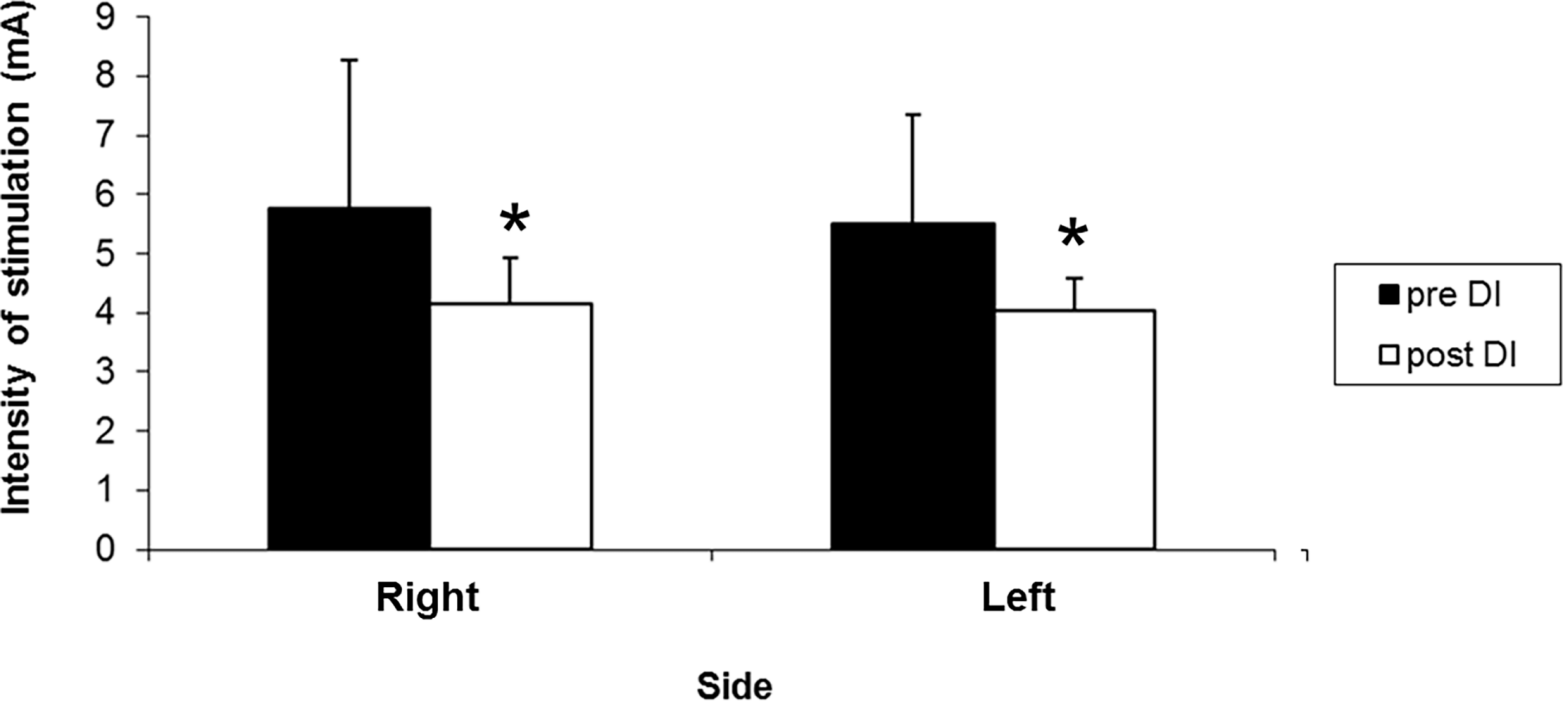
Sensory thresholds in mA (n = 12) before and after dry immersion (Right z = -2.54; p = 0.011; Left z = -3.059; p = 0.002).

We found a significant decrease in sensory thresholds after DI (-20.75 +/-21.7%; z = -2.54; p = 0.0109 on the right side and -22.18+/-17.28%; z = -3.059; p = 0.002 on the left side) (Fig 3).

### SEPs

**Fig 4:**
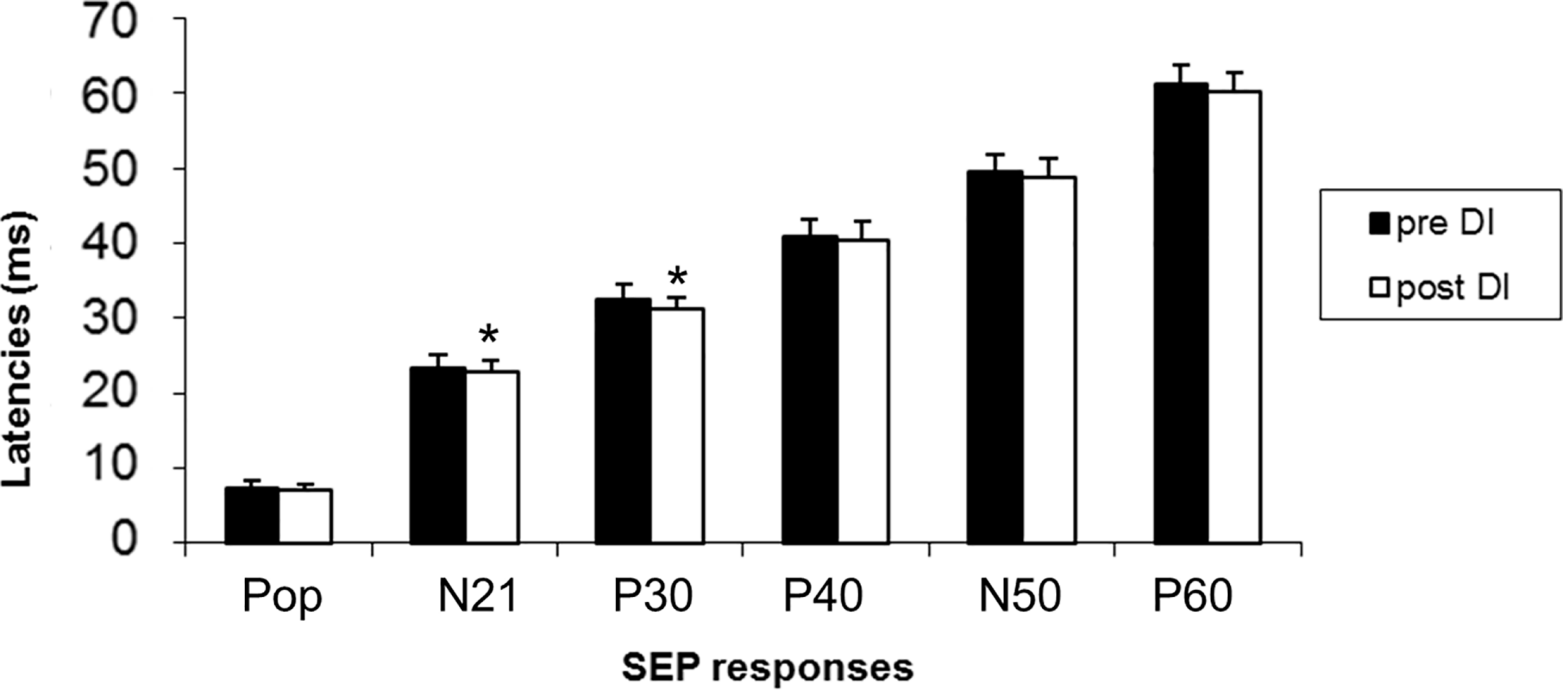
Mean latencies of SEP responses after stimulation of the right tibial nerve before and after 3 days of dry immersion.

**Fig 5:**
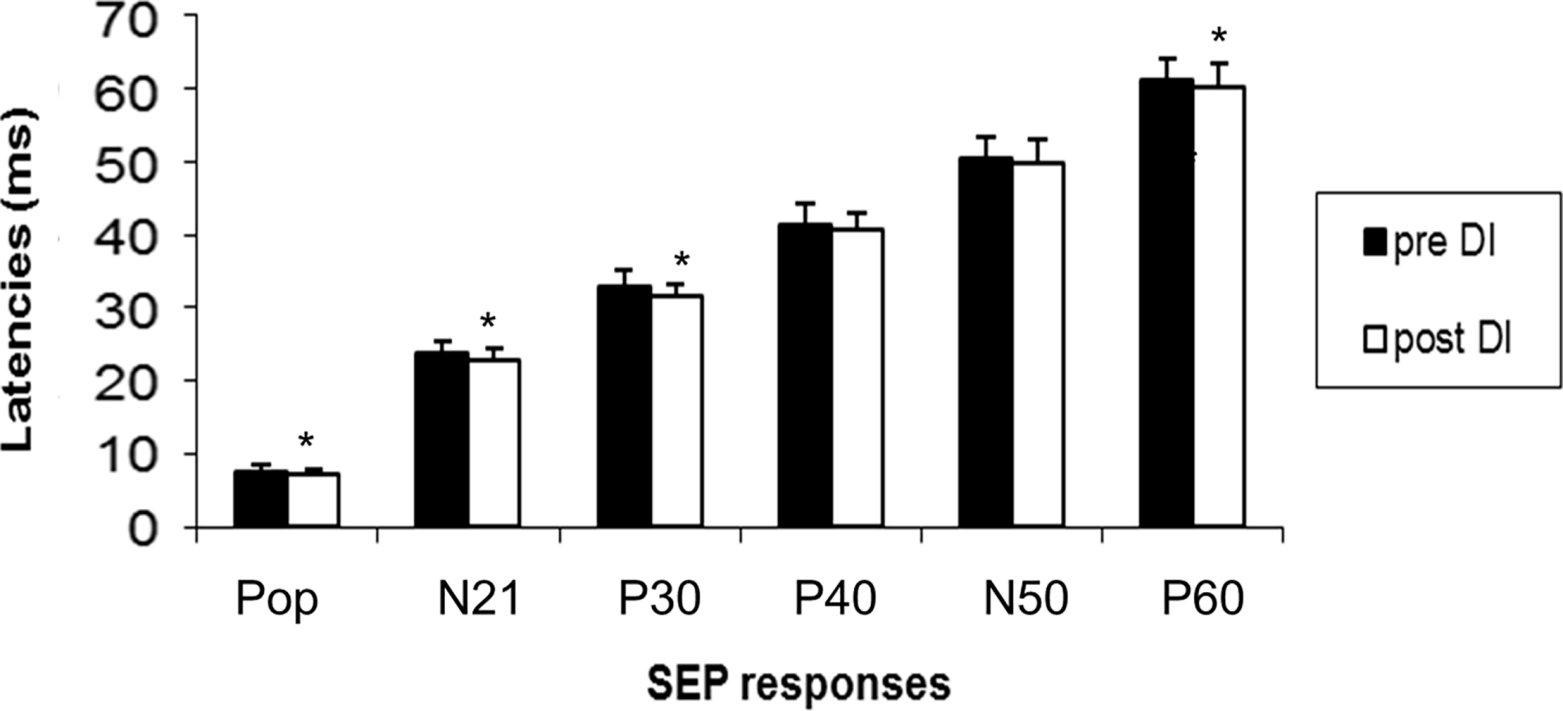
Mean latencies of SEP responses after stimulation of the left tibial nerve before and after 3 days of dry immersion.

**Fig 6:**
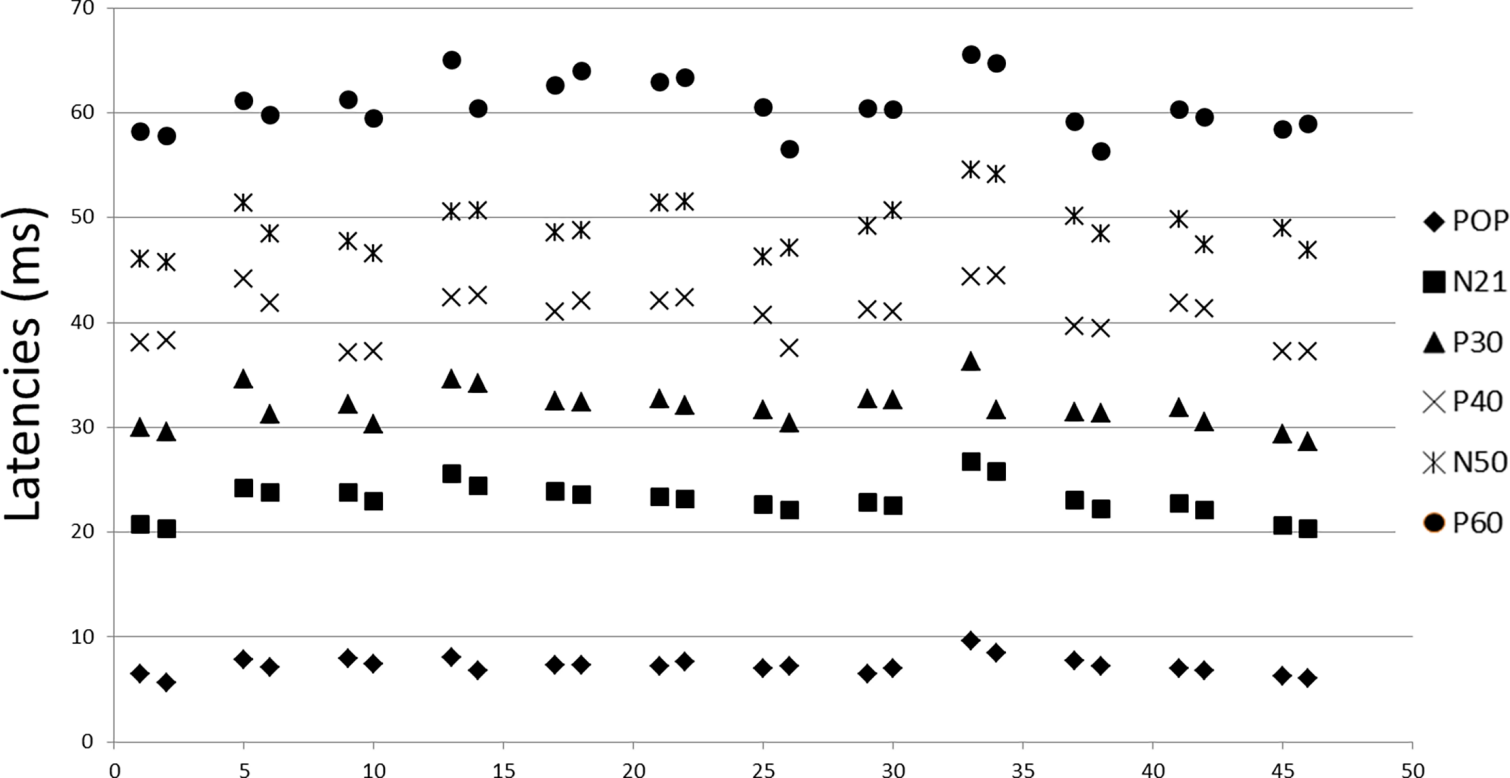
Latencies of SEP responses after stimulation of the right tibial nerve before and after 3 days of dry immersion. Individual raw data.

**Fig 7:**
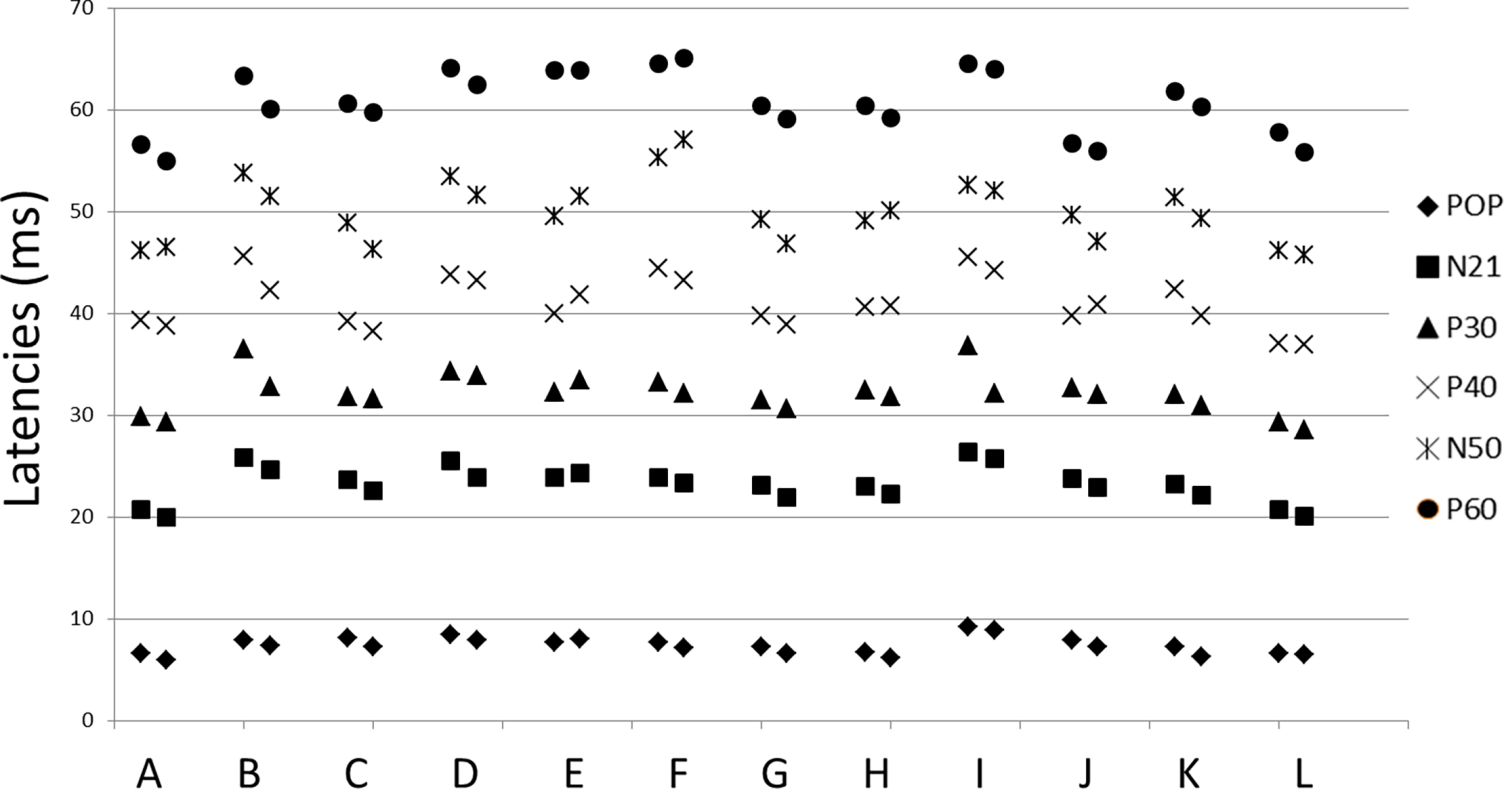
Latencies of SEP responses after stimulation of the left tibial nerve before and after 3 days of dry immersion. Individual raw data.

Concerning the amplitude of P40, we found no significant difference between the baseline and DI data. We neither found any significant difference between the baseline and DI data pour N50-P60 amplitude. Popliteal response latencies were shortened on the left side (0.5+/-0.36; z = -2.86; p = 0.003), and tended to be shorter on the right side, but they were not statistically significant (0.36+/-0.55; z = -1.86; p = 0.06). Latencies of all the central responses until the P30 were shortened after DI compared to baseline (N21 right:-0.57+/-0.31; z = -3.06; p = 0.002; N21 left -0.83+/-0.53; z = -2.94; p = 0.003; P30 right: -1.26+/-1.42; z = -3.059; p = 0.002; P30 left: -1.11+/-1.55; z = -2.27; p = 0.02). Other cortical responses were not different, except P60 on the left side (-1.16+/-0.99; z = -2.8; p = 0.005) (Figs 4 and 5). At the individual level, these results were homogeneous (Table 1, Figs 6 and 7).

We did not find any difference between peripheral, medullar or central conduction time between baseline and after the third day of DI.

**Table 1:** Mean latencies of the SEP responses before and after DI.

SEP responses		Right (mean latency and standard deviation)	Left (mean latency and standard deviation)
Popliteal	Pre	7.41+/-0.9	7.65+/-0.8
	post	7.05+/-0.7	7.15+/-0.86
	z (p)	-1.86 (p = 0.06)	**-2.86 (p = 0.003)**
N21	Pre	23.32+/-1.74	23.69+/-1.74
	post	22.78+/-1.57	22.86+/-1.73
	z (p)	**-3.06 (p = 0.002)**	**-2.94 (p = 0.003)**
P30	Pre	32.51+/-1.93	32.76+/-2.26
	post	31.25+/-1.49	31.65+/-1.56
	z (p)	**-3.059 (p = 0.002)**	**-2.27 (p = 0.022)**
P40	Pre	40.82+/-2.44	41.48+/-2.80
	post	40.43+/-2.44	40.78+/-2.27
	z (p)	-0.445 (p = 0.655)	-1.56 (p = 0.11)
N50	Pre	49.54+/-2.34	50.46+/-2.90
	post	48.87+/-2.44	49.64+/-3.34
	z (p)	-1.49 (p = 0.13)	-1.76 (p = 0.13)
P60	Pre	61.29+/-2.36	61.23+/-2.98
	post	60.10+/-2.73	60.08+/-3.34
	z (p)	-1.88 (p = 0.059)	**-2.8 (p = 0.005)**

We found no correlation between the decrease in sensory thresholds and electrophysiological data.

## Discussion

### Sensory thresholds

A statistically significant decrease in sensory thresholds was found after three days of DI. The decrease in sensory thresholds is in accordance with the study by Lowrey et al. Anecdotal observational studies suggest that the sole of the foot becomes hypersensitive following space flight. In their study, Lowrey et al. measured the skin sensitivity of eleven astronauts by vibration perception at the great toe, fifth metatarsal and heel. Data were collected pre- and post-flight. They found a decrease in skin sensitivity on the landing day at 3 Hz and 25 Hz on the great toe. Also, six astronauts presented increased sensitivity at 250 Hz at the heel [12]. However, these results were not confirmed later, and Strzalkowski et al. found that short duration space flight increases the low frequency (3 Hz) perception threshold [13].

### SEPs

An acceleration of the conduction at the initial part of the lemniscal pathway until the cervicobulbar response was found after three days of DI.

Regarding the decrease in SEP mean latencies, this was mainly explained by a decrease in the early latencies up to P 30, but did not significantly affect cortical responses; the P30 response is mainly generated by the activity of the bulbothalamic neuron of the lemniscal pathway. This may be explained by the fact that, in SEPs interpretation, a dysfunction at a precise level does not systematically induce a modification of the responses generated downstream from the lesion[14], especially when the volley is only dispersed by a demyelinating process. In this case a component can be abnormal in latency or amplitude, but may not impact later responses because of synaptic synchronization [15]. In our study, synaptic synchronization occurs at the bulbothalamic neuron level and can explain the fact that cortical responses are not modified. This could explain why we only found a decrease in the early responses induced by DI, but no impact on cortical responses.

This hyperexcitability could be explained by a modification of the expression of membranous channels caused by deafferentation. Prolonged hind limb unloading leads to changes in electrophysiological properties of L5 dorsal root ganglion neurons in rats after 14 days [16]. The shedding induces a decrease in the rheobase and excitability thresholds of these neurons. This could be linked to variations of ionic currents, which could be due to an increase in the expression of membranous sodium channels or to a decrease in potassium conductance. However, in these studies a deceleration of the sensory conduction is usually noticed, but these modifications were observed following 14 days of deafferentation, when structural demyelination signs may occur secondary to neuronal hyperexcitability [16, 17].

Medullar plasticity could also be involved. Some key points related to sensory processing by the spinal cord are as follows: (1) within the musculoskeletal and cutaneous tissues is an extensive network of mechanoreceptors and metaboreceptors that continuously update the spinal cord on the physiologic state of the peripheral tissues; (2) these receptors provide an ensemble of highly integrated and perceptually meaningful information to the spinal cord; (3) the spinal cord is smart, as demonstrated by its ability to interpret and appropriately respond to highly complex and meaningful sensoryensembles; and (4) the human spinal cord demonstrates this smartness and automaticity [18]. It is well accepted that there exists short-term task dependant modulation of spinal reflexes and this modulation does not immediately impose any structural or long lasting functional change in spinal circuits. The prevailing notion is that the synaptic strength is alterned in a task specific manner [19].In animals, cat studies using distal cutaneous denervation showed increased magnitude of the responses evoked by stimulation of the motor cortex in unaffected proximal muscles suggesting incresed corticospinal efficacy [20]. Diminishing afferent input by dorsal rootlet rhizotoy in adults rats led to a significant increase in corticospinal connections, including those on cholinergic interneurons [21]. Hence we think that altering sensory afferences input could modify spinal circuitry responses.

These phenomena have been well studied to explain phantom limb pain triggered by amputation. The lack of peripheral afferents affects neurons of the dorsal horn of the medulla and modifies the structural conformation of the synapses. This process is called central sensitization and includes several phenomena. The neurons of the dorsal horns become hyperexcitable and a reduction of inhibitory processes occurs with the release of glutamate and neurokinins. It is associated with an anatomical reorganization of the sensory inputs in the laminae. The loss of the afferent influx can also provoke disinhibition of the medulla, with a decrease in GABAergic activity and a down-regulation of the opioid receptors of the dorsal horns [1, 22].

Some studies using DI also found medullar hyperreflexia [3]. It would be interesting in further studies to complete this neurophysiological evaluation by a measurement of the F waves and H reflexes to prove this hypothesis.

However we can not rule out an involvement of an alteration upstream to the popliteal site (the most peripherical site studied): skin, fiber endings, or very peripherical part of nerve fibers. We did not controlled skin temperature but the experimental conditions were really reproducible. We did not find any data in the litterature to support this hypothesis.

### Inflight experiments

Inflight experiments have demonstrated that the absence of gravity modifies the stimuli associated with proprioception and suggestthat microgravity could impair the state of the proprioceptive sensory receptors; neuromuscular spindles, Golgi tendon organs, tactile receptors and joint receptors, as a result of atrophy of the antigravity muscles and fluid shifts, or the sudden release of a constant muscle tone. Daily sleep on Earth however does not impair those mechanoreceptors. The virtual absence of gravity modifies the stimuli associated with proprioception and impacts knowledge of limb position [23]. During the Spacelab-1 flight objective measurement of limb position awareness was carried out by asking crew members to point at target positions with their eyes closed, both inflight and immediately postflight. Results of this experiment indicated that pointing accuracy was degraded during and immediately after flight [24]. Many studies have dealt with the effect of microgravity on these basic mechanisms involving proprioceptive information processing. However, most of them have specifically focused on one of these mechanisms, using either a reflexological approach to analyze spinal excitability [18, 25–27] or vestibulospinal reactivity [28, 29], or psychophysiological methods to study higher cognitive functions such as body or limb movement perception [30–33] or body representation in terms of systems of coordinates [34, 35]. The “illusions” experiment carried out on five astronauts during two Franco-Russian flights (Antarès 1992 and Altair 1993) and in the Russian post-Antares mission aimed at investigating the adaptive changes in human proprioceptive functions occurring in weightlessness at both the sensorimotor and cognitive levels, focusing on whole body postural reflexes and whole body movement perception[36]. The nature of these changes suggests that new relationships between proprioceptive sensory afferents and motor command are set up in weightless environments, which involves a high degree of sensorimotor adaptability[36] The second point concerns the finding that proprioceptive feedback contributes less efficiently to postural regulation during the early post-flight period, as demonstrated by the gradual increase in the amplitude of the vibration-induced postural responses recorded during the first week [36]. A large depression of the cortical response to vibration in weightlessness compared to normogravity was observed [37].

In contrast to these studies, our work suggests an enhancement of sensitivity to proprioceptive inputs. However, dry immersion is a model of weightlessness with suppression of the proprioceptive afferents, but in this model the vestibular afferents were not affected, contrary to microgravity. To our knowledge SEPs during space flight have never been evaluated and do not allow direct comparison with our results. Our results could be explained by early compensation strategies in response to the lack of proprioceptive afferents.

## Conclusion

Three days of dry immersion are sufficient to induce a significant decrease in sensory thresholds associated with a shortening of SEP latencies, which may reflect hyperexcitability of lemniscal pathways. Further studies are needed to confirm this hyperexcitability and to clarify whether it is transient or not.

## Supporting information

S1 Table General data. (DOCX)

S2 Table Sensory thresholds before and after DI. Individual data.
(DOCX)

S3 Table Latencies of the SEP popliteal and lumbar responses before and after DI. Individual data.
(DOCX)

S4 Table Latencies of the SEP cortical responses before and after DI. Individual data. (DOCX)

S5 Table Amplitude of the SEP cortical responses(N30-P40) before and after DI. Individual data. (DOCX)

S6 Table Amplitude of the SEP cortical responses (N50-P60) befoire and after DI. Individual mean data.
(DOCX)
