# Supplementary material for: Dry immersion as a model of deafferentation: A neurophysiology study using somatosensory evoked potentials
Source: PLoS One. 2018 Aug 22;13(8):e0201704. doi: 10.1371/journal.pone.0201704 (PMC6104952; doi:10.1371/journal.pone.0201704)
Supplement: S1 Table — (DOCX) [file pone.0201704.s001.docx]

S1 Table : General data

|  | Age | Height (m) | Weight (kg) | BMI |
| --- | --- | --- | --- | --- |
| A | 39 | 1,67 | 68,5 | 24,5616551 |
| B | 39 | 1,83 | 87,3 | 26,0682612 |
| C | 34 | 1,72 | 76 | 25,6895619 |
| D | 36 | 1,83 | 71 | 21,2009914 |
| E | 33 | 1,84 | 73,8 | 21,7982042 |
| F | 26 | 1,74 | 71,8 | 23,7151539 |
| G | 30 | 1,74 | 67,5 | 22,294887 |
| H | 28 | 1,82 | 80,8 | 24,3931892 |
| I | 34 | 1,89 | 86,6 | 24,2434422 |
| J | 26 | 1,78 | 70,5 | 22,2509784 |
| K | 30 | 1,8 | 77,1 | 23,7962963 |
| L | 26 | 1,69 | 67 | 23,4585624 |
