# Supplementary material for: Dry immersion as a model of deafferentation: A neurophysiology study using somatosensory evoked potentials
Source: PLoS One. 2018 Aug 22;13(8):e0201704. doi: 10.1371/journal.pone.0201704 (PMC6104952; doi:10.1371/journal.pone.0201704)
Supplement: S2 Table — Individual data. (DOCX) [file pone.0201704.s002.docx]

S2 Table : Sensory thresholds before and after DI. Individual data

|  | Sensory threshold (ST) PRE right (mA) | ST POST Right (mA) | DELTA ST Right (%) | ST PRE Left (mA) | ST POST Left (mA) | DELTA ST Left (%) |
| --- | --- | --- | --- | --- | --- | --- |
| A | 4,10 | 3,70 | 9,76 | 4,50 | 4,20 | 6,67 |
| B | 12,00 | 4,50 | 62,50 | 10,00 | 4,20 | 58,00 |
| C | 4,50 | 4,50 | 0,00 | 7,20 | 4,20 | 41,67 |
| D | 4,00 | 3,40 | 15,00 | 3,90 | 3,70 | 5,13 |
| E | 4,50 | 4,70 | -4,44 | 4,50 | 4,30 | 4,44 |
| F | 5,00 | 5,00 | 0,00 | 4,50 | 3,20 | 28,89 |
| G | 4,00 | 3,40 | 15,00 | 4,50 | 3,40 | 24,44 |
| H | 4,00 | 4,00 | 0,00 | 4,30 | 4,00 | 6,98 |
| I | 7,50 | 5,30 | 29,33 | 7,50 | 4,50 | 40,00 |
| J | 9,00 | 5,00 | 44,44 | 5,00 | 4,50 | 10,00 |
| K | 5,90 | 3,30 | 44,07 | 6,00 | 5,00 | 16,67 |
| L | 4,50 | 3,00 | 33,33 | 4,30 | 3,30 | 23,26 |
| Mean | 5,75 | 4,15 | 20,75 | 5,52 | 4,04 | 22,18 |
| Standard deviation | 2,52028137 | 0,78102497 | 21,7290362 | 1,83592104 | 0,54515775 | 17,2877113 |
