# Supplementary material for: Dry immersion as a model of deafferentation: A neurophysiology study using somatosensory evoked potentials
Source: PLoS One. 2018 Aug 22;13(8):e0201704. doi: 10.1371/journal.pone.0201704 (PMC6104952; doi:10.1371/journal.pone.0201704)
Supplement: S3 Table — Individual data. (DOCX) [file pone.0201704.s003.docx]

S3 Table: Latencies of the SEP popliteal and lumbar responses before and after DI. Individual data.

|  | POP PRE Right (R) | POP POST R | DELTA POP R | POP PRE Left (L) | POP POST L | DELTA POP L | N21 PRE R | N21 POST R | DELTA N21 R | N21 PRE L | N21 POST L | DELTA N21 L |
| --- | --- | --- | --- | --- | --- | --- | --- | --- | --- | --- | --- | --- |
| A | 6,50 | 5,60 | 13,85 | 6,60 | 6,00 | 9,09 | 20,70 | 20,30 | 1,93 | 20,80 | 20,00 | 3,85 |
| B | 7,80 | 7,10 | 8,97 | 7,90 | 7,40 | 6,33 | 24,20 | 23,80 | 1,65 | 25,90 | 24,70 | 4,63 |
| C | 7,90 | 7,40 | 6,33 | 8,20 | 7,30 | 10,98 | 23,80 | 23,00 | 3,36 | 23,70 | 22,60 | 4,64 |
| D | 8,10 | 6,80 | 16,05 | 8,50 | 7,90 | 7,06 | 25,60 | 24,40 | 4,69 | 25,60 | 23,90 | 6,64 |
| E | 7,30 | 7,30 | 0,00 | 7,70 | 8,10 | -5,19 | 23,90 | 23,60 | 1,26 | 23,90 | 24,40 | -2,09 |
| F | 7,20 | 7,60 | -5,56 | 7,70 | 7,20 | 6,49 | 23,40 | 23,20 | 0,85 | 23,90 | 23,40 | 2,09 |
| G | 7,00 | 7,20 | -2,86 | 7,30 | 6,70 | 8,22 | 22,60 | 22,10 | 2,21 | 23,20 | 22,00 | 5,17 |
| H | 6,50 | 7,00 | -7,69 | 6,80 | 6,20 | 8,82 | 22,80 | 22,50 | 1,32 | 23,00 | 22,30 | 3,04 |
| I | 9,60 | 8,50 | 11,46 | 9,30 | 8,90 | 4,30 | 26,70 | 25,80 | 3,37 | 26,40 | 25,80 | 2,27 |
| J | 7,70 | 7,20 | 6,49 | 7,90 | 7,30 | 7,59 | 23,10 | 22,20 | 3,90 | 23,80 | 22,90 | 3,78 |
| K | 7,00 | 6,80 | 2,86 | 7,30 | 6,30 | 13,70 | 22,70 | 22,10 | 2,64 | 23,30 | 22,20 | 4,72 |
| L | 6,30 | 6,10 | 3,17 | 6,60 | 6,50 | 1,52 | 20,60 | 20,30 | 1,46 | 20,80 | 20,10 | 3,37 |
| Mean | 7,41 | 7,05 | 4,42 | 7,65 | 7,15 | 6,58 | 23,34 | 22,78 | 2,39 | 23,69 | 22,86 | 3,51 |
| Standard deviation (SD) | 0,90599853 | 0,7242551 | 7,5379387 | 0,80056798 | 0,8639234 | 4,81326809 | 1,74379854 | 1,5760278 | 1,20495611 | 1,74640323 | 1,73543724 | 2,17260285 |
