# Supplementary material for: Dry immersion as a model of deafferentation: A neurophysiology study using somatosensory evoked potentials
Source: PLoS One. 2018 Aug 22;13(8):e0201704. doi: 10.1371/journal.pone.0201704 (PMC6104952; doi:10.1371/journal.pone.0201704)
Supplement: S4 Table — (DOCX) [file pone.0201704.s004.docx]

S4 Table: Latencies of the SEP cortical responses before and after DI. Individual data.

|  | P30 PRE R | P30 POST R | DELTA P30L | P30 PRE L | P30 POST L | DALTA P30L | P40 PRE R | P40 POST R | DELTA P40R | P40 PRE L | P40 POST L | DELTA P40L | N50 PRE R | N50 POST R | DELTA N50 R | N50 PRE L | N50 POST L | DELTA N50L | P60 PRE R | P60 POST R | DELTA P60R | P60 PRE L | P60 POST L | DELTA P60L |
| --- | --- | --- | --- | --- | --- | --- | --- | --- | --- | --- | --- | --- | --- | --- | --- | --- | --- | --- | --- | --- | --- | --- | --- | --- |
| A | 30,00 | 29,60 | 1,33 | 29,90 | 29,30 | 2,01 | 38,10 | 38,30 | -0,52 | 39,30 | 38,80 | 1,27 | 46,00 | 45,70 | 0,65 | 46,20 | 46,50 | -0,65 | 58,20 | 57,80 | 0,69 | 56,60 | 55,00 | 2,83 |
| B | 34,60 | 31,20 | 9,83 | 36,50 | 32,80 | 10,14 | 44,20 | 41,80 | 5,43 | 45,70 | 42,30 | 7,44 | 51,40 | 48,40 | 5,84 | 53,80 | 51,50 | 4,28 | 61,10 | 59,80 | 2,13 | 63,40 | 60,10 | 5,21 |
| C | 32,20 | 30,30 | 5,90 | 31,90 | 31,60 | 0,94 | 37,10 | 37,20 | -0,27 | 39,20 | 38,30 | 2,30 | 47,70 | 46,60 | 2,31 | 48,90 | 46,30 | 5,32 | 61,30 | 59,50 | 2,94 | 60,60 | 59,80 | 1,32 |
| D | 34,60 | 34,20 | 1,16 | 34,30 | 33,90 | 1,17 | 42,40 | 42,60 | -0,47 | 43,80 | 43,30 | 1,14 | 50,50 | 50,70 | -0,40 | 53,50 | 51,60 | 3,55 | 65,00 | 60,40 | 7,08 | 64,10 | 62,50 | 2,50 |
| E | 32,50 | 32,40 | 0,31 | 32,30 | 33,50 | -3,72 | 41,00 | 42,00 | -2,44 | 40,00 | 41,80 | -4,50 | 48,60 | 48,80 | -0,41 | 49,60 | 51,50 | -3,83 | 62,60 | 64,00 | -2,24 | 63,90 | 63,90 | 0,00 |
| F | 32,70 | 32,10 | 1,83 | 33,30 | 32,20 | 3,30 | 42,10 | 42,40 | -0,71 | 44,40 | 43,30 | 2,48 | 51,40 | 51,50 | -0,19 | 55,30 | 57,10 | -3,25 | 62,90 | 63,40 | -0,79 | 64,50 | 65,10 | -0,93 |
| G | 31,70 | 30,40 | 4,10 | 31,50 | 30,70 | 2,54 | 40,70 | 37,50 | 7,86 | 39,80 | 38,90 | 2,26 | 46,30 | 47,10 | -1,73 | 49,20 | 46,80 | 4,88 | 60,50 | 56,50 | 6,61 | 60,40 | 59,10 | 2,15 |
| H | 32,70 | 32,60 | 0,31 | 32,50 | 31,90 | 1,85 | 41,20 | 41,00 | 0,49 | 40,70 | 40,80 | -0,25 | 49,20 | 50,70 | -3,05 | 49,10 | 50,10 | -2,04 | 60,40 | 60,30 | 0,17 | 60,40 | 59,20 | 1,99 |
| I | 36,30 | 31,70 | 12,67 | 36,80 | 32,20 | 12,50 | 44,40 | 44,50 | -0,23 | 45,50 | 44,20 | 2,86 | 54,50 | 54,10 | 0,73 | 52,60 | 52,10 | 0,95 | 65,60 | 64,70 | 1,37 | 64,60 | 64,00 | 0,93 |
| J | 31,50 | 31,40 | 0,32 | 32,70 | 32,10 | 1,83 | 39,60 | 39,40 | 0,51 | 39,80 | 40,90 | -2,76 | 50,10 | 48,50 | 3,19 | 49,70 | 47,10 | 5,23 | 59,20 | 56,30 | 4,90 | 56,70 | 56,00 | 1,23 |
| K | 31,90 | 30,50 | 4,39 | 32,10 | 31,00 | 3,43 | 41,80 | 41,30 | 1,20 | 42,40 | 39,80 | 6,13 | 49,80 | 47,40 | 4,82 | 51,40 | 49,30 | 4,09 | 60,30 | 59,60 | 1,16 | 61,80 | 60,30 | 2,43 |
| L | 29,40 | 28,60 | 2,72 | 29,30 | 28,60 | 2,39 | 37,20 | 37,20 | 0,00 | 37,10 | 37,00 | 0,27 | 49,00 | 46,90 | 4,29 | 46,20 | 45,80 | 0,87 | 58,40 | 58,90 | -0,86 | 57,80 | 55,90 | 3,29 |
| Mean | 32,51 | 31,25 | 3,74 | 32,76 | 31,65 | 3,20 | 40,82 | 40,43 | 0,90 | 41,48 | 40,78 | 1,55 | 49,54 | 48,87 | 1,34 | 50,46 | 49,64 | 1,62 | 61,29 | 60,10 | 1,93 | 61,23 | 60,08 | 1,91 |
| SD | 1,9374108 | 1,49939382 | 3,98262556 | 2,25970969 | 1,56175892 | 4,24210019 | 2,43752234 | 2,44255207 | 2,86857159 | 2,80101443 | 2,26869339 | 3,29788001 | 2,34577312 | 2,44404037 | 2,7522057 | 2,90405933 | 3,34486264 | 3,40048581 | 2,36160898 | 2,73396149 | 2,96003915 | 2,98399773 | 3,34123684 | 1,58837133 |
