# Supplementary material for: Dry immersion as a model of deafferentation: A neurophysiology study using somatosensory evoked potentials
Source: PLoS One. 2018 Aug 22;13(8):e0201704. doi: 10.1371/journal.pone.0201704 (PMC6104952; doi:10.1371/journal.pone.0201704)
Supplement: S5 Table — (DOCX) [file pone.0201704.s005.docx]

S5 Table: Amplitude of the SEP cortical responses(N30-P40) before and after DI. Individual data.

|  | Amplitude N30-P40 pre Right (R) 1 | Amplitude  N30-P40  Pre R2 | Mean amplitude N30-P40 pre R | Amplitude N30-P40 pre Left (L)1 | Amplitude N30-P40 pre L2 | Mean amplitude N30-P40 pre L | Amplitude  N30-P40 post R1 | Amplitude N30-P40 post R2 | Mean amplitude  N30-P40 postR | Amplitude N30-P40 post L1 | Amplitude N30-P40 post L2 | Mean amplitude N30-P40  postL |
| --- | --- | --- | --- | --- | --- | --- | --- | --- | --- | --- | --- | --- |
| A | 2,15 | 2,16 | 2,155 | 2,49 | 3,21 | 2,85 | 2,45 | 2,64 | 2,545 | 2,12 | ? | 2,12 |
| B | 1,31 | 0,76 | 1,035 | 0,5 | ? | 0,5 | 0,86 | 0,786 | 0,823 | 0,77 | 1,05 | 0,91 |
| C | 0,78 | 0,91 | 0,845 | 1,65 | 1,49 | 1,57 | 0,92 | 1,02 | 0,97 | 0,97 | 1,1 | 1,035 |
| D | 2,25 | 1,81 | 2,03 | 1,31 | 1,9 | 1,605 | 1,62 | 1,98 | 1,8 | 1,3 | 1,14 | 1,22 |
| E | 2,98 | ? | 2,98 | 2,38 | 1,45 | 1,915 | 2,65 | 1,32 | 1,985 | 1,19 | 1,81 | 1,5 |
| F | 3,55 | 3,16 | 3,355 | 3,36 | 3,15 | 3,255 | 3,72 | 3,52 | 3,62 | 2,85 | ? | 2,85 |
| G | 1,16 | 1,12 | 1,14 | 0,34 | 0,61 | 0,475 | 1,7 | 2,38 | 2,04 | 1,01 | 0,87 | 0,94 |
| H | 0,81 | 0,82 | 0,815 | 1,88 | 1,92 | 1,9 | 0,57 | 1,45 | 1,01 | 1,1 | 1,37 | 1,235 |
| I | 1,06 | 1,26 | 1,16 | 1,17 | 1,03 | 1,1 | 2,6 | 1,74 | 2,17 | 1,66 | 2,37 | 2,015 |
| J | 4,16 | 3,51 | 3,835 | 2,68 | 2,15 | 2,415 | 3,88 | 3,38 | 3,63 | 2,32 | 1,07 | 1,695 |
| K | 0,81 | 0,69 | 0,75 | 1,76 | 2,24 | 2 | 1,52 | 0,8 | 1,16 | 1,73 | 1,22 | 1,475 |
| L | 1,19 | 1,11 | 1,15 | 0,9 | 1,55 | 1,225 | 1,19 | 0,67 | 0,93 | 1,07 | 1,21 | 1,14 |
| Mean |  |  | 1,77083333 |  |  | 1,73416667 |  |  | 1,89025 |  |  | 1,51125 |
| SD |  |  | 1,08535883 |  |  | 0,8509963 |  |  | 0,99001094 |  |  | 0,57725103 |
