# Supplementary material for: Dry immersion as a model of deafferentation: A neurophysiology study using somatosensory evoked potentials
Source: PLoS One. 2018 Aug 22;13(8):e0201704. doi: 10.1371/journal.pone.0201704 (PMC6104952; doi:10.1371/journal.pone.0201704)
Supplement: S6 Table — Individual mean data. (DOCX) [file pone.0201704.s006.docx]

S6 Table: Amplitude of the SEP cortical responses (N50-P60) befoire and after DI. Individual mean data.

|  | Mean amplitude N50-P60 pre R | Mean amplitude N50-P60  pre L | Mean amplitude  N50-P40 postR | Mean amplitude N50-P60  postL |
| --- | --- | --- | --- | --- |
| A | 3,1 | 3,5 | 4,2 | 2,8 |
| B | 1,76 | 0,76 | 1,78 | 1,39 |
| C | 4,9 | 4 | 3,5 | 3,1 |
| D | 2,6 | 1,96 | 2,1 | 1,83 |
| E | 4 | 4,1 | 2,7 | 2,1 |
| F | 5,5 | 1,64 | 4,8 | 0,71 |
| G | 2,1 | 1,86 | 2,3 | 2,9 |
| H | 3,3 | 3,5 | 2,4 | 2,3 |
| I | 3,5 | 1,98 | 3,8 | 3 |
| J | 3,1 | 1 | 1,5 | 1,79 |
| K | 3,5 | 3 | 3,2 | 3,5 |
| L | 1,8 | 3,2 | 0,78 | 2,1 |
| Mean | 3,26333333 | 2,54166667 | 2,755 | 2,29333333 |
| SD | 1,15149968 | 1,14883208 | 1,17961858 | 0,80321324 |
